# Visual analysis of obesity and hypothyroidism: A bibliometric analysis

**DOI:** 10.1097/MD.0000000000036841

**Published:** 2024-01-05

**Authors:** Lanying Yao, Long Zhang, Yuxing Tai, Rongsheng Jiang, Jianzhong Cui, Xiaochao Gang, Mingjun Liu

**Affiliations:** a Changchun University of Chinese Medicine, Changchun Jilin, Nanguan District, China.

**Keywords:** bibliometric analysis, current state of research, hypothyroidism, obesity

## Abstract

**Background:**

The prevalence of obesity is gradually increasing and is closely associated with hypothyroidism. It is of research interest to explore the association between obesity and hypothyroidism and the current status of research.

**Methods:**

We chose the Web of Science Core Collection (WoSCC) database as the data source and searched to obtain relevant literature on obesity and hypothyroidism. And we used CiteSpace and VOSviewer to analyze the related literature.

**Results:**

A total of 508 articles were included in the literature, with an overall increasing trend in the number of publications. There were 170 relevant countries or organizations, and the United States was the country with the most publications. There were 1742 related organizations, and the Egyptian Knowledge Bank (EKB) was the organization with the most publications. There are 3015 authors involved, and there is a clear collaboration between authors. There are 227 related journals and J CLIN ENDOCR METAB is the most cited journal. The most frequently occurring keywords were obesity and hypothyroidism, but also other related topics such as bariatric surgery, metabolic syndrome, insulin resistance, body mass index, and leptin.

**Conclusion:**

The research related to obesity and hypothyroidism is gradually gaining attention, and the research direction is gradually expanding to metabolic syndrome, insulin resistance, leptin, and other related topics.

## 1. Introduction

Obesity is a public health epidemic^[1]^ that negatively affects the health of patients. The prevalence of obesity has gradually increased with changes in the social environment and eating habits. As early as 2008, the number of obese adults in the United States had reached 930,000 people^[2]^ In 2008, the number of obese adults in the U.S. reached 930,000 people. Some studies predict that the prevalence of obesity will increase by 33% in 2030, with the prevalence of severe obesity increasing by 130%.^[3]^ The prevalence of severe obesity will increase by 130%. With the increasing prevalence of obesity, the adverse effects on human life are becoming more and more significant, and it has become a worldwide health problem.

As research on obesity continues to progress, scholars have found that obesity is closely associated with a variety of diseases^[4–6]^ hypothyroidism is a typical obesity-associated disease. Hypothyroidism is a typical obesity-associated disease.^[7,8]^ and there is a 2-way influence between the 2^[9]^ A systematic review and meta-analysis A systematic review and meta-analysis showed that obesity is significantly associated with hypothyroidism and suggested that prevention of obesity is essential for thyroid disease.^[10]^ A large cohort study was also conducted. A large cohort study also showed a high correlation between obesity metabolic abnormalities and hypothyroidism in men.^[11]^ Further studies have found that bariatric surgery improves thyroid function and medication dosage in patients with hypothyroidism.^[12,13]^ Although scholars have conducted numerous studies to explore the biological mechanisms between obesity and hypothyroidism, it is not yet fully understood.^[14,15]^ Therefore, summarizing the current research status of obesity and hypothyroidism is of great significance in controlling the future research direction and expanding the therapeutic methods.

Bibliometrics allows the application of mathematical and statistical methods for quantitative analysis of relevant knowledge carriers. As a comprehensive body of knowledge, bibliometrics allows for the statistical analysis and visualization of literature^[16]^ Bibliometrics can be used to analyze and visualize literature statistically. Currently, bibliometrics has been widely used in several research fields. The purpose of this study is to conduct a systematic and comprehensive statistical analysis of the literature related to obesity and hypothyroidism published in the Web of Science database in recent years, applying 2 visualization tools, CiteSpace and VOSviewer, to draw a knowledge map, and analyze the trend of literature publication, countries, authors, research institutions, keywords and co-citations. To summarize the evolution of research hotspots and research trends related to obesity and hypothyroidism in recent years.

## 2. Materials and methods

### 2.1. Search strategy

We searched the WoSCC for literature on obesity and hypothyroidism. The search terms included “Hypothyroidism” and synonyms, “obesity” and synonyms, and so on. The publication period of the literature was limited to 2022. We finally retrieved 1277 kinds of literature. The specific literature search strategy is shown in Table 1.

**Table 1:** Literature retrieval strategies.

ID	Results	Search strategy
#1	23355	TS=(“Hypothyroidism” OR “Hypothyroidisms” OR “Thyroid-Stimulating Hormone Deficiency” OR “Deficiency, Thyroid-Stimulating Hormone” OR “Hormone Deficiency, Thyroid-Stimulating” OR “Thyroid Stimulating Hormone Deficiency” OR “Thyroid-Stimulating Hormone Deficiencies” OR “TSH Deficiency” OR “Deficiency, TSH” OR “TSH Deficiencies” OR “Secondary Hypothyroidism” OR “Hypothyroidism, Secondary” OR “Secondary Hypothyroidisms”)
#2	441108	TS = (“obesity” OR “obese*” OR “overweight”)
#3	1277	#1AND#2

### 2.2. Inclusion criteria

We included relevant literature published in different academic journals related to obesity and hypothyroidism. The language of the articles was limited to English, the type of literature was limited to ARTICLE and REVIEW, and we did not limit the species under study.

### 2.3. Exclusion criteria

We will exclude clinical guidelines, letters, conference abstracts, editorials, dissertations, biographies, book reviews, conference presentations, news reports, duplicate publications, publications withdrawn by the authors, etc, and literature under topics that are not medically relevant will also be excluded.

### 2.4. Literature screening process

**Figure 1.**
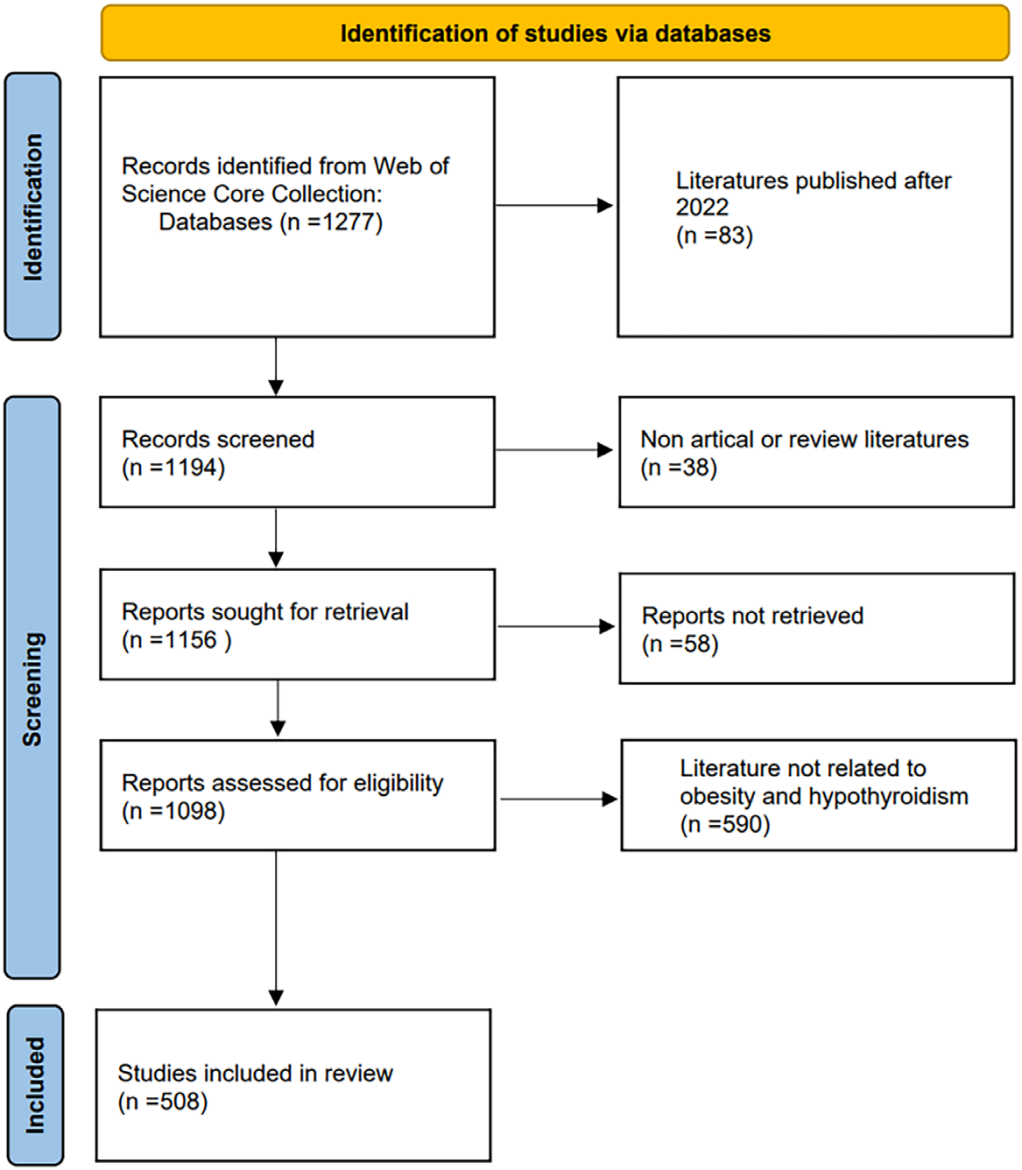
Documents screening process.

Two researchers screened the literature based on the screening results and the inclusion and exclusion criteria, and 508 articles were finally included. The specific screening process is shown in Figure 1.

### 2.5. Bibliometric analysis software

We used 2 software programs, CiteSpace and VOSviewer, for bibliometric and visualization analyses, and we focused on collaborative relationships in the research in this field, as well as co-occurring relationships between authors, keywords, countries, research institutes, and network diagrams.

## 3. Results

### 3.1. Publication trends

**Figure 2.**
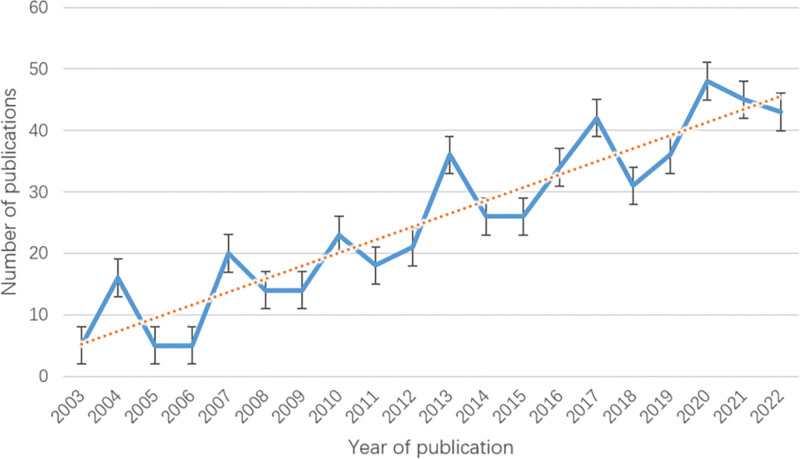
Trends in publication volume.

Figure 2 shows the trend in publications between 2003 and 2022, with an overall upward trend. 2005 to 2006, 2008 to 2009, and 2014 to 2015 were flat, with no significant fluctuations. 2012 saw an upward trend in the number of publications, but the overall number of publications was relatively low. 2012 saw a significant increase in the number of publications, but there were still significant fluctuations. increased, but there were still more pronounced fluctuations. Our linear regression model predicts (y = 2.1278x + 3.0579 R² = 0.8561) that the field is currently in a phase of steady growth in global publication output.

### 3.2. Country or area analysis

**Figure 3.**
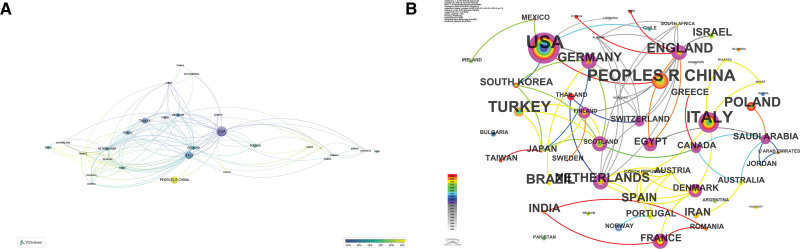
(A) Co occurrence diagram of national or regional cooperative relationships. (B) The central position of a country or region.

A total of 170 countries or regions were involved in the 508 papers, and the top 10 countries in terms of the number of papers published are shown in Table 2. In Figure 3A, we analyzed the 30 countries or regions with at least 5 publications to form a collaborative network graph. The node color indicates clustering, the size of the node indicates the number of articles, and the connecting line between the nodes indicates the strength of collaboration. Among them, the United States published the largest number of relevant papers with 96 articles and a total link strength of 62. Italy had 63 articles and a total link strength of 50. China followed with 59 articles and a total link strength of 4. However, in terms of the centrality data, the centrality of the Netherlands, the United Kingdom, Germany, and Switzerland was greater than that of the United States. This suggests that although the United States has published the most literature, it has not yet been able to develop an absolute lead in terms of influence. Figure 3B illustrates the centrality between countries or regions, with the area of the outer circle of the node indicating the centrality of the region. It can be visualized that the United States, Italy, China, and the United Kingdom are at the center of research in this area.

**Table 2:** Ten countries or regions with the highest number of documents issued.

Rank	Country or regions	Publication number	Centrality	Total link strength
1	USA	96	0.3	62
2	ITALY	63	0.12	50
3	PEOPLES R CHINA	59	0.00	4
4	TURKEY	43	0.00	16
5	ENGLAND	27	0.54	16
6	BRAZIL	26	0.00	61
7	POLAND	26	0.00	8
8	NETHERLANDS	25	0.6	35
9	GERMANY	24	0.42	45
10	INDIA	20	0.00	4

### 3.3. Institutional analysis

**Figure 4.**
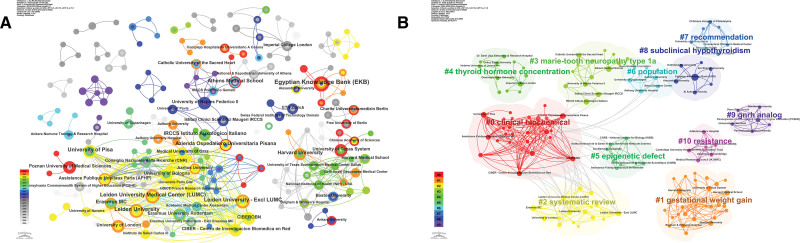
(A) The central position of the institution. (B) Cluster analysis diagram between institutions.

There are 1742 organizations related to publications, and Table 3 shows the organizations with more than 5 publications. We base our analysis on the number of publications issued. The area of the outer circle of the nodes in Figure 4A indicates the centrality of the area. It can be seen that the institution with the most number of articles is the EKB, and the second and third are Universidade do Estado do Rio de Janeiro and China Medical University, respectively. Figure 4B shows the results of the cluster analysis among institutions, and the different color areas representing different combinations. It can be seen that these institutions can form 10 clusters, which indicates that there is relatively close cooperation between institutions.

**Table 3:** Institutions with the highest volume of documents issued.

Rank	Count	Centrality	Institutions
1	10	0	Egyptian Knowledge Bank (EKB)
2	9	0	Universidade do Estado do Rio de Janeiro
3	8	0	China Medical University
4	7	0.01	University of Pisa
5	7	0.01	Leiden University Medical Center (LUMC)
6	7	0.01	Leiden University - Excl LUMC
7	7	0.01	Leiden University
8	6	0	Harvard University
9	6	0.01	Erasmus MC
10	6	0.01	Athens Medical School
11	6	0.03	Azienda Ospedaliero Universitaria Pisana
12	6	0	IRCCS Istituto Auxologico Italiano

Combining the analysis of institutions with centrality, the first place in centrality is Azienda Ospedaliero Universitaria Pisana. University of Naples Federico II, Assistance Publique Hopitaux Paris (APHP), and Aarhus University have the same centrality and are in 2nd place.Azienda Ospedaliero Universitaria Pisana has 6 publications each.The University of Naples Federico II, Assistance Publique Hopitaux Paris (APHP) Paris (APHP), and Aarhus University have 5, 5, and 4 publications respectively. The centrality of the institution is proportional to the number of publications.

### 3.4. Author analysis

#### 3.4.1. Analysis of author collaboration.

**Figure 5.**
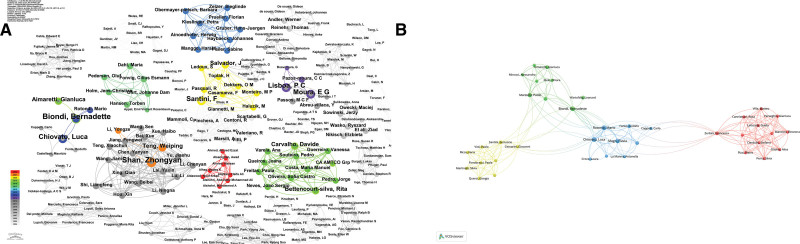
(A) Author Collaboration Network Diagram. (B) Author Cluster Analysis Chart.

According to our search results, a total of 3015 authors have published literature related to obesity and hypothyroidism. Table 4 demonstrates the top 12 authors in terms of the number of publications, and each author published at least 5 papers. Figure 5A illustrates a network diagram of author collaborations constructed based on the number of publications per author. Among all the authors, Lisboa, P. C. and Moura, E. G. are the 2 authors with the highest number of publications, and the collaboration between the 2 scholars is also more prominent, with both of them having a total link strength of 45. There is also a strong relationship between the 3 groups of scholars, namely, Chiovato, Luca and Biondi, Bernadette, Shan, Zhongyan and Teng, Weiping, and the 3 groups of scholars, Chiovato, Luca and Biondi, Bernadette, and Shan, Zhongyan and Teng, Weiping. Weiping3 groups of scholars also have stronger collaborations between them. Among all the collaborations, 3 Chinese scholars, Gao, Ling, Zhao, Jiajun, and Shan, Zhongyan, have the top 3 total link strengths of 63, 63, and 62, respectively, and each of them has published 7 related papers. Figure 5B, on the other hand, shows the results of the cluster analysis among authors with high publication volume, with different colored regions representing different combinations. These authors formed 4 different clusters, and the larger nodes in each cluster indicate that the team has more publications.

**Table 4:** Authors with the highest number of posts.

Rank	Author	Documents	Citations	Total link strength
1	Lisboa, P. C.	8	185	45
2	Moura, E. G.	8	185	45
3	Gao, Ling	7	139	63
4	Shan, Zhongyan	7	206	62
5	Teng, Weiping	7	216	47
6	Zhao, Jiajun	7	139	63
7	Chiovato, Luca	6	371	34
8	Rotondi, Mario	6	371	34
9	Biondi, Bernadette	5	408	22
10	Magri, Flavia	5	221	28
11	Reinehr, Thomas	5	696	0
12	Song, Yongfeng	5	116	46

#### 3.4.2. Analysis of co-cited authors.

**Figure 6.**
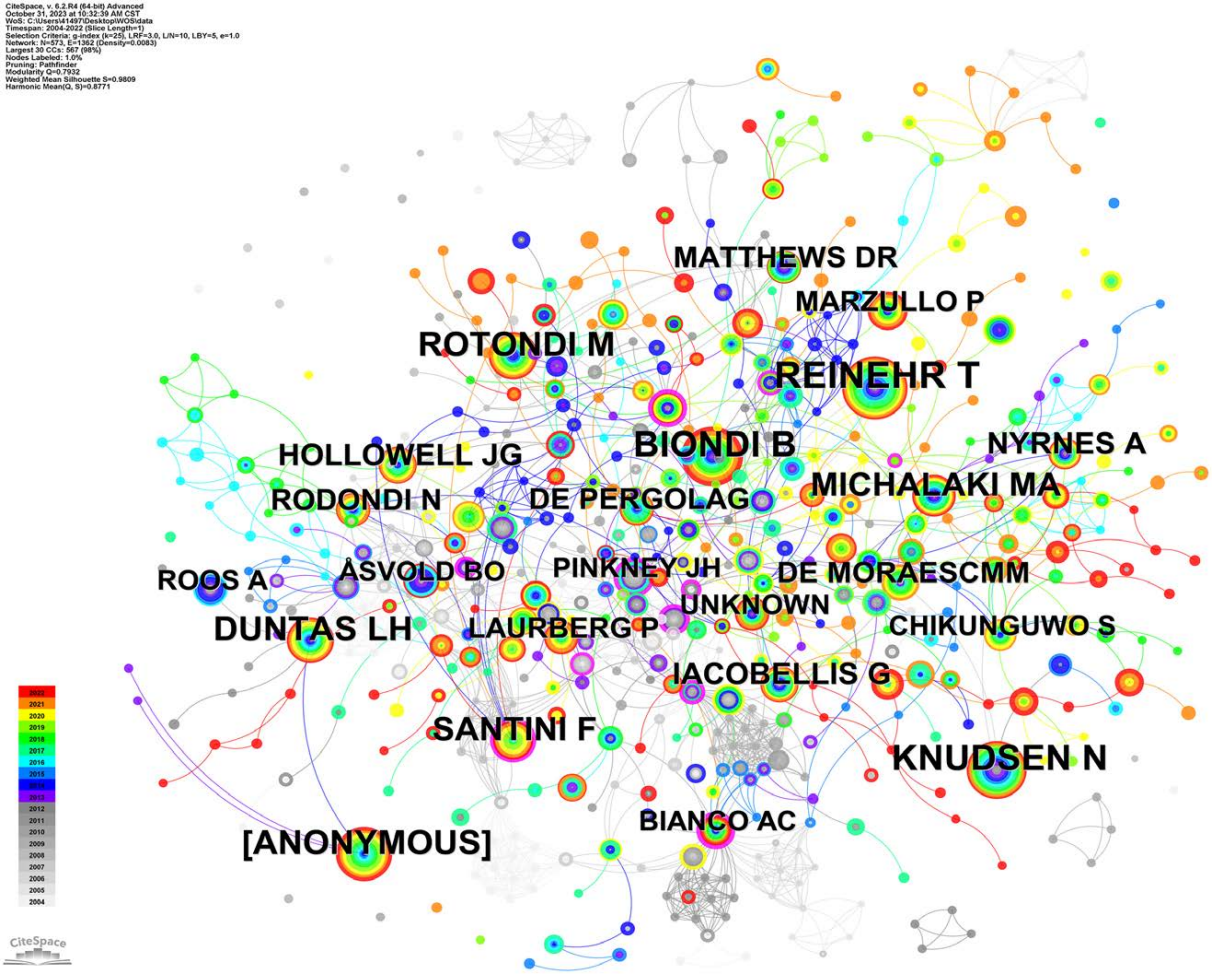
Co-citation author co-occurrence analysis chart.

We show the number of co-citations and centrality of the top 10 authors in Table 5. Figure 6 shows the results of the co-occurrence analysis of the co-cited authors. It can be found that REINEHR T has the highest number of citations with 119, but the centrality is 0.01. Although DUNTAS LH, MICHALAKI MA, ROTONDI M, and HOLLOWELL JG have only 74, 68, 74, and 68 publications, their centrality is 0.05, 0.04, 0.03, and 0.03, respectively. Although these 4 authors are not in the absolute leading position in terms of the number of publications, they have also produced a large impact, indicating the high quality of their research.

**Table 5:** Co-citation of the highest authors.

Rank	Count	Centrality	Yr	Cited authors
1	119	0.01	2006	REINEHR T.
2	99	0.01	2006	KNUDSEN N.
3	97	0.02	2007	BIONDI B.
4	79	0.01	2011	ANONYMOUS
5	74	0.05	2004	DUNTAS L.H.
6	74	0.03	2010	ROTONDI M.
7	68	0.04	2008	MICHALAKI M.A.
8	65	0.11	2005	SANTINI F.
9	48	0.03	2006	HOLLOWELL J.G.
10	45	0.02	2007	NYRNES A.

Among all the co-cited authors, the top 3 authors in terms of centrality are AHIMA RS (0.28), PINKNEY JH (0.25), and BIANCO AC (0.24), but their publications are only 17, 30, and 31, respectively. the articles of these 3 authors are of high quality and recognized by scholars of the related fields.

### 3.5. Journal analysis

**Figure 7.**
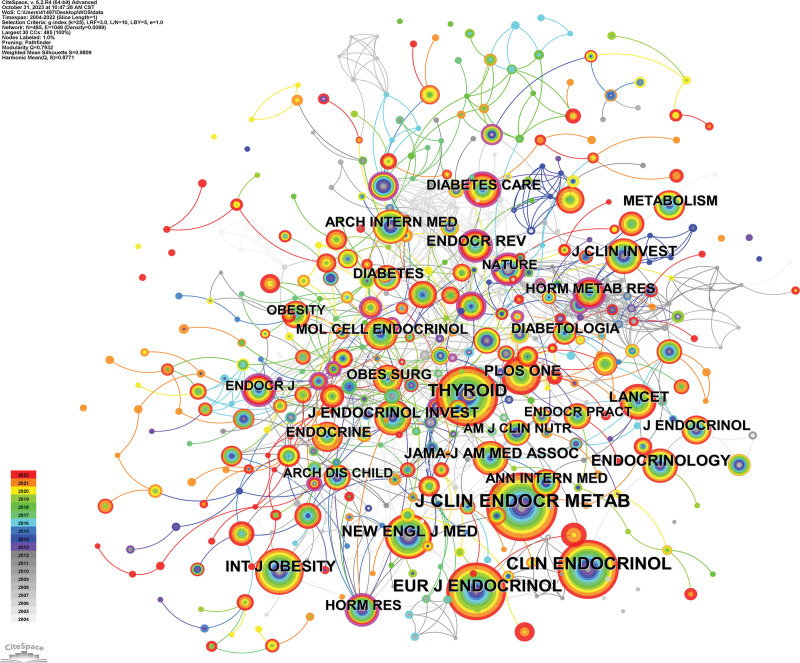
Co occurrence analysis chart of cited journals.

A total of 227 journals published literature related to obesity and hypothyroidism. Table 6 demonstrates the top 10 most cited journals with centripetal and journal division, impact factors. Figure 7 demonstrates the cited journals, where the top 3 cited journals are J CLIN ENDOCR METAB, CLIN ENDOCRINOL, and THYROID, which have been cited 414, 317, and 315 times, respectively. Combined with the impact factor and JCR partition, the latest impact factor of J CLIN ENDOCR METAB is 5.8 points, the JCR partition is Q1, the latest impact factor of CLIN ENDOCRINOL is 3.2 points, and the JCR partition is Q3, and the latest impact factor of THYROID is 6.6, and the JCR partition is Q1. 10 journals include the top journals of the field of Medicine The top journals of medical field NEW ENGL J MED, JAMA-J AM MED ASSOC, LANCET, it can be seen that the issue of obesity and hypothyroidism has been paid attention by the researchers and there are high-quality literature published with authority.

**Table 6:** The most cited periodicals.

Rank	Count	Centrality	Cited	JCR	IF
1	414	0	J CLIN ENDOCR METAB	Q1	5.8
2	317	0.02	CLIN ENDOCRINOL	Q3	3.2
3	315	0	THYROID	Q1	6.6
4	293	0.01	EUR J ENDOCRINOL	Q1	5.8
5	182	0.02	INT J OBESITY	Q2	4.9
6	180	0.03	NEW ENGL J MED	Q1	158.5
7	156	0.01	ENDOCRINOLOGY	Q2	4.8
8	147	0.02	JAMA-J AM MED ASSOC	Q1	120.7
9	145	0.02	LANCET	Q1	168.9
10	138	0.03	J ENDOCRINOL INVEST	Q2	5.4

### 3.6. Analysis of references

**Figure 8.**
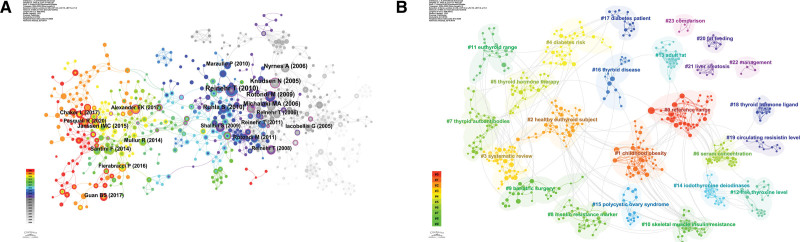
(A) Co-citation and co-occurrence analysis chart. (B) Cluster analysis of co-cited literature.

Table 7 demonstrates the most co-cited literature. The network map of co-cited literature presented in Figure 8A was performed based on the references of 508 papers. There are a total of 690 co-cited references in the 508 papers related to obesity and hypothyroidism in the last 20 years. Table 7 demonstrates the top 10 co-cited literature. One of the most cited literature was published in Molecular and cellular endocrinology. Figure 8B demonstrates the results of the cluster analysis of the co-cited literature, which shows a clearer theme.

**Table 7:** Most cited documents.

Rank	Count	Centrality	Yr	Cite references
1	29	0.11	2010	Reinehr T, 2010, MOL CELL ENDOCRINOL, V316, P165, DOI 10.1016/j.mce.2009.06.005
2	19	0.11	2009	Rotondi M, 2009, EUR J ENDOCRINOL, V160, P403, DOI 10.1530/EJE-08-0734
3	18	0.12	2005	Knudsen N, 2005, J CLIN ENDOCR METAB, V90, P4019, DOI 10.1210/jc.2004-2225
4	18	0.08	2006	Michalaki MA, 2006, THYROID, V16, P73, DOI 10.1089/thy.2006.16.73
5	18	0.02	2010	Ruhla S, 2010, CLIN ENDOCRINOL, V72, P696, DOI 10.1111/j.1365-2265.2009.03698.x
6	16	0.04	2006	Nyrnes A, 2006, INT J OBESITY, V30, P100, DOI 10.1038/sj.ijo.0803112
7	14	0	2015	Janssen IMC, 2015, SURG OBES RELAT DIS, V11, P1257, DOI 10.1016/j.soard.2015.02.021
8	14	0	2014	Santini F, 2014, EUR J ENDOCRINOL, V171, PR137, DOI 10.1530/EJE-14-0067
9	13	0.1	2005	Iacobellis G, 2005, CLIN ENDOCRINOL, V62, P487, DOI 10.1111/j.1365-2265.2005.02247.x
10	13	0.05	2010	Marzullo P, 2010, J CLIN ENDOCR METAB, V95, P3965, DOI 10.1210/jc.2009-2798

### 3.7. Keyword analysis

#### 3.7.1. Keyword co-occurrence analysis.

**Figure 9.**
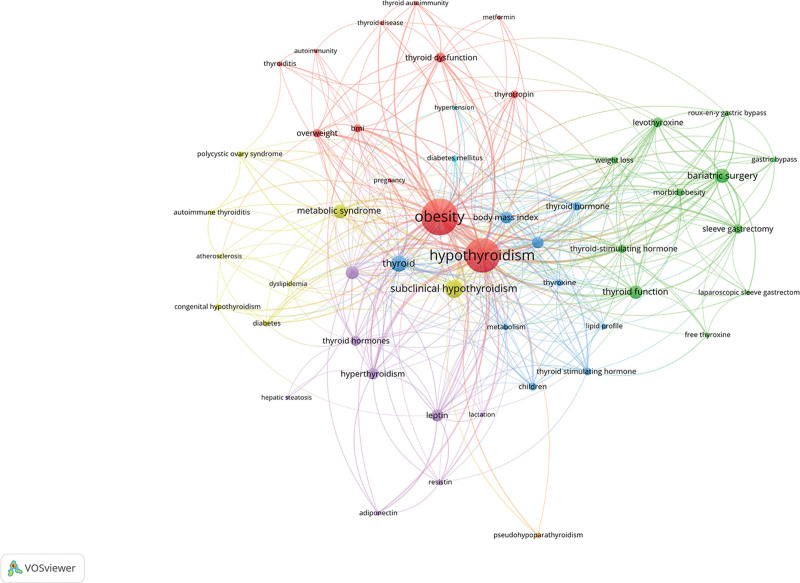
Keyword co-occurrence analysis chart.

Table 8 shows the keywords with the top 10 occurrences, with the most occurrences being obesity, which also ranks first with a total link strength of 292. Among all the keywords, there are 12 occurrences more than 20 times. We applied VOSviewer to analyze the co-occurrence of the keywords, there are 51 keywords with at least 5 occurrences, and the larger the area of the node indicates more occurrences, as shown in Figure 9.

**Table 8:** Keywords with the highest frequency.

Rank	Keyword	Occurrences	Total link strength
1	Obesity	142	292
2	Hypothyroidism	127	256
3	Subclinical hypothyroidism	45	77
4	Thyroid	38	85
5	Bariatric surgery	32	81
6	Metabolic syndrome	30	48
7	Thyroid function	29	54
8	Insulin resistance	27	68
9	Body mass index	24	61
10	Leptin	24	57

#### 3.7.2. Keyword clustering analysis.

**Figure 10.**
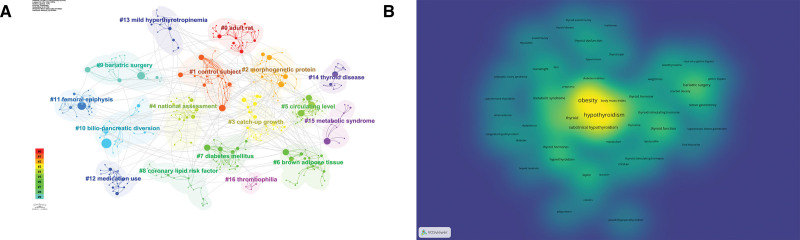
(A) Cluster analysis graph of keywords. (B) Heat map of keywords.

Figure 10A shows the cluster analysis of the keywords, which can be categorized into multiple clusters. Figure 10B shows the heat map analysis of the keywords, with the words Obesity and hypothyroidism at the core.

#### 3.7.3. Keyword timeline analysis.

**Figure 11.**
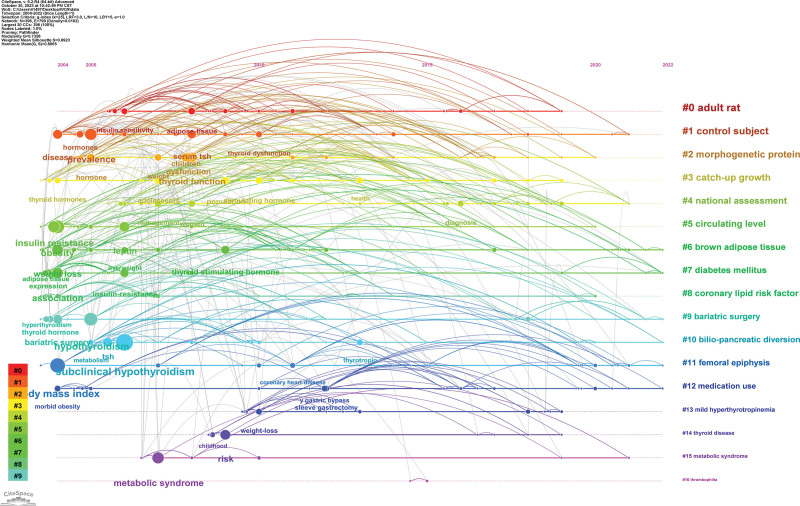
Timeline visualization analysis of keywords.

We further analyze these keywords. Figure 11 shows a timeline visualization of the keywords, reflecting the evolution of the keywords. It can be seen that scholars initially focused on the topics of “subclinical hypothyroidism,” “insulin resistance,” and “weight loss.” weight loss,” and by 2010, “thyroid function” and “thyroid-stimulating hormone” became the hot topics of research. In 2010, “thyroid function” and “thyroid-stimulating hormone” became the hot topics of research. Currently, these topics continue to receive focused attention from scholars.

## 4. Discussion

Based on a search of the WOS core library, we conducted an econometric analysis of the literature related to obesity and hypothyroidism to summarize the relevant information and research status of studies in this area. We initially retrieved 1277 documents, which were manually screened to improve the accuracy of the study, and finally, 508 documents were screened for inclusion in this study.

The analysis of countries and regions shows that the United States, Italy, and China have published the most literature in this field. At the same time, the Netherlands, the United Kingdom, Germany, and Switzerland have a strong centrality, while there is a cooperative relationship between several countries. Research in this area has been emphasized by mainstream countries.

In the results of the authorship profile analysis, Lisboa, P. C., and Moura, E. G. were the authors with the highest number of publications. A close collaborative relationship exists between the 2 authors, whose research focuses on the association between obesity and thyroid function in women during pregnancy and the postpartum period^[17,18]^ The third most widely published author is Dr K. K. The third most widely published author is Chinese scholar Gao, Ling, who conducted a large cohort study and proposed that modification of the AMPK/PPARγ/GPAT3 axis through TSH receptors may serve as a potential therapeutic target for obesity.^[11,19]^ Another Chinese scholar, Shan, Shan, and Shan, Shan, is the author of the study. Another Chinese scholar, Shan, Zhongyan, also conducted a large cohort study to investigate the association between obesity and different metabolic phenotypes of thyroid disease in Chinese adults.^[20]^ The results were analyzed by the corresponding authors. Among the results of the analysis of co-cited authors, REINEHR T has the most citations with 119. His high-impact literature exploring the prevalence and clinical manifestations of definable somatic diseases in overweight children is highly influential.^[21]^ The link between obesity and hypothyroidism can be traced back to the maternal period and it is important to conduct large cohort studies to explore the link between the 2. Meanwhile, scholars have also explored the biological mechanism between obesity and hypothyroidism.

A total of 227 journals published literature related to obesity and hypothyroidism, of which 3 journals, CLIN ENDOCR METAB, CLIN ENDOCRINOL, and THYROID, were the most cited journals. In terms of JCR partitioning, J CLIN ENDOCR METAB and THYROID are both in Q1, while CLIN ENDOCRINOL is in Q3. In terms of impact factor, THYROID had an impact factor of 6.6 points, J CLIN ENDOCR METAB was 5.8 points and CLIN ENDOCRINOL was 3.2 points. However, it was also found that 3 top journals, NEW ENGL J MED, JAMA-J AM MED ASSOC, and LANCET, are also highly cited journals. It can be seen that the specialized and top journals in this field have received a lot of attention from scholars and have been heavily cited.

An analysis of the references revealed 508 documents with 690 co-references and the highest number of citations at 29, published in Molecular and Cellular Endocrinology. This document focuses on the link between obesity and thyroid function and suggests that leptin affects the release of thyroid hormones.^[22]^ The results of cluster analysis of the co-cited literature revealed that these papers could be clustered into 24 combinations with clear themes.

The results of the keyword analysis show the topics that scholars focus on in this field, and without a doubt, the most frequent occurrences are obesity and hypothyroidism. among the top 10 occurrences of keywords, we also found bariatric surgery, metabolic syndrome, insulin resistance, body mass index, and leptin, which are all keywords that are closely related to the topics of obesity and hypothyroidism. Cluster analysis revealed that the keywords could be clustered into 16 combinations with strong correlations. The timeline analysis demonstrated the change of research themes among scholars, who initially focused on “subclinical hypothyroidism,” “insulin resistance” and “weight loss,” but in recent years, scholars have focused on “obesity” and “hypothyroidism.” Initially, scholars focused on “subclinical hypothyroidism,” “insulin resistance,” and “weight loss,” but in recent years, “thyroid function” and “thyroid-stimulating hormone” have become hot topics of research. In recent years, “thyroid function” and “thyroid-stimulating hormone” have become hot topics of research.

## 5. Restrictions

Although we tried to maintain the rigor of this study as much as possible, there are still some limitations due to some objective reasons. Due to the software, we only included articles from the WOS core library and literature from other high-quality databases could not be included. Due to the extensive nature of the search measurement strategy, the initial search results were not very satisfactory, so we manually screened the results of the initial search, which made the included literature somewhat subjective. Therefore, the results of this study only represent the results of the analysis of the included literature.

## 6. Conclusion

The association between obesity and hypothyroidism will gradually receive more attention, and its related research topics will gradually expand. Scholars are gradually shifting from describing the association between the 2 to explaining the mechanisms and causes of the 2. Meanwhile, relevant research results are gradually published in high-quality journals, forming a certain influence. Based on the research results of obesity and hypothyroidism, the association between hypothyroidism and metabolic syndrome, insulin resistance, and leptin will also receive attention.

## Acknowledgments

We thank those who participated in and supported this study.

## Author contributions

**Conceptualization:** Lanying Yao, Mingjun Liu.

**Data curation:** Rongsheng Jiang, Jianzhong Cui, Xiaochao Gang.

**Formal analysis:** Jianzhong Cui.

**Investigation:** Long Zhang, Rongsheng Jiang.

**Methodology:** Lanying Yao.

**Supervision:** Yuxing Tai, Mingjun Liu.

**Validation:** Yuxing Tai, Long Zhang, Xiaochao Gang.

**Visualization:** Yuxing Tai.

**Writing – original draft:** Lanying Yao.

**Writing – review & editing:** Long Zhang, Mingjun Liu.
